# Durability of protection of ancestral-strain COVID-19 third- and fourth-dose vaccine boosters against Omicron XBB/XBB.1 and JN.1 symptomatic infection, hospitalisation and mortality in Indonesian adults (2023–2024): a test-negative case–control study

**DOI:** 10.1016/j.lansea.2025.100689

**Published:** 2025-11-01

**Authors:** Bayu Satria Wiratama, Vicka Oktaria, Khoriah Indrihutami, Muhammad Hardhantyo, Ahmad Watsiq Maula, Hanevi Djasri, Rizka Dinari, Muhammad Syairaji, Suwarti, Ariel Pradipta, Dwi Utomo Nusantara, Ardiana Kusumaningrum, Andrew, Marillyn Mariana Tamburian, Ilma Safira Baehaqi, Rebecca Merrill, Henry Surendra, Fetty Wijayanti, Benjamin A. Dahl, Raph L. Hamers

**Affiliations:** a Department of Biostatistics, Epidemiology, and Population Health, Faculty of Medicine, Public Health, and Nursing, Universitas Gadjah Mada, Yogyakarta, Indonesia; b Center for Health Policy and Management, Faculty of Medicine, Public Health, and Nursing, Universitas Gadjah Mada, Yogyakarta, Indonesia; c Center for Child Health - Pediatric Research Office, Faculty of Medicine, Public Health, and Nursing, Universitas Gadjah Mada, Yogyakarta, Indonesia; d Oxford University Clinical Research Unit Indonesia, Faculty of Medicine, Universitas Indonesia, Jakarta, Indonesia; e Faculty of Health Science, Universitas Respati Yogyakarta, Yogyakarta, Indonesia; f Department of Health Information and Services, Vocational College, Universitas Gadjah Mada, Yogyakarta, Indonesia; g Genomik Solidaritas Indonesia Lab, Jakarta, Indonesia; h Department of Biochemistry and Molecular Biology, Faculty of Medicine, Universitas Indonesia, Jakarta, Indonesia; i Pasar Minggu Regional Hospital, Jakarta, Indonesia; j University of Indonesia Hospital, Depok, Indonesia; k Department of Clinical Microbiology, Faculty of Medicine, Universitas Indonesia, Jakarta, Indonesia; l Tugu Koja Regional Hospital, Jakarta, Indonesia; m Country Office, Centers for Disease Control and Prevention, Jakarta, Indonesia; n Monash University Indonesia, Tangerang, Indonesia; o Global Immunization Division, Global Health Center, Centers for Disease Control and Prevention, Atlanta, USA; p Centre for Tropical Medicine and Global Health, Nuffield Department of Medicine, University of Oxford, Oxford, UK

**Keywords:** COVID-19, SARS-CoV-2 Omicron subvariants, Vaccine boosters, Vaccine effectiveness, Vaccine waning, Long-term immunity, Post-pandemic, Comorbidities, Indonesia, Test-negative design

## Abstract

**Background:**

SARS-CoV-2 ancestral-strain vaccines have effectively reduced SARS-CoV-2-related severe illness and death worldwide. However, waning immunity over time has warranted revaccination to boost immunity. Indonesia, like most low- and middle-income countries, has not provided regular vaccine boosters post-pandemic. This study assessed the longer-term durability of protection from ancestral-strain third- and fourth-dose boosters.

**Methods:**

We conducted a test-negative case–control study among symptomatic adults seeking SARS-CoV-2 testing at 14 purposely selected test sites in the major cities of Yogyakarta and Jakarta (March 2023–May 2024). Test-positive individuals were cases and test-negative individuals were controls. SARS-CoV-2 variants were identified using whole genome sequencing. We used multivariable logistic regression to estimate absolute or incremental vaccine effectiveness (VE) against symptomatic infection and COVID-19-related hospitalisation or death, adjusted for main confounders.

**Findings:**

Of 2439 participants (median age 35 years, 56.2% female), 388 were cases and 2051 controls. Vaccination with two primary doses, a third-dose or fourth-dose booster did not provide sustained protection against Omicron XBB/JN.1 symptomatic infection up to median 27, 20 or 13 months since administration, respectively. However, there was sustained incremental protection from the third-dose booster (administered median 20 month prior) against hospitalisation (VE 38.3% [95% CI 3.9–60.3]) and death (55.2% [17.7–75.6]) for older individuals (aged >50 years), and against death (55.2% [12.8–76.9]) for individuals with one or more comorbidities. There was also sustained incremental protection from the fourth-dose booster (administered median 13 months prior) against hospitalisation for older individuals (50.2% [10.3–72.3]) and individuals with one or more comorbidities (74.4% [49.2–87.1]).

**Interpretation:**

Ancestral-strain vaccine boosters provided durable, moderate protection against severe or fatal outcomes from Omicron XBB/JN.1 infection for older and comorbid individuals. The findings highlight the benefits of improving access to revaccination for vulnerable groups in Indonesia.

**Funding:**

US Centers for Diseases Control and Prevention.

**Research in context**

**Evidence before this study**

We searched PubMed on April 1, 2025, for articles on the effectiveness of ancestral-strain vaccine boosters against Omicron and subvariants, using the search terms (“SARS-CoV-2” OR “COVID-19”) AND (“vaccine effectiveness” OR “vaccine booster”) AND (“death” OR “mortality” OR “disease severity” OR “hospitalisation”) AND (“Omicron”). A hospital-based cohort study in Indonesia, spanning 2020–2023, found that individuals who had received at least one vaccine dose (mostly CoronaVac), after median six months since last dose, had a 98% reduced risk of severe clinical disease and 56% reduced risk of death from Omicron BA.1/BA.2, BA.4/BA.5 and XBB subvariants, compared with unvaccinated individuals. Studies mostly from high-income countries in North America, Europe and Asia indicated that ancestral-strain booster vaccines provide moderate protection against severe and fatal outcomes from Omicron subvariants, typically waning after six months, especially against recent immune-evasive subvariants like XBB or JN.1.

**Added value of this study**

This multicentre, test-negative case–control study, conducted in two major Indonesian cities, is among the few studies in low- and middle-income countries (LMICs) that evaluated the longer-term durability of protection of third- and fourth-dose ancestral-vaccine vaccine boosters after primary vaccination with inactivated whole-virus vaccines, against recent Omicon subvariants. This study thus addresses gaps in existing evidence that is predominantly from populations in high-income countries. Ancestral-strain mRNA or viral vector heterologous third-dose and fourth-dose vaccine boosters were found to maintain a durable moderate protective effect (up to 20 and 13 months, respectively) against severe or fatal outcomes from Omicron XBB/JN.1 sub-variants for older adults (over 50 years of age) and individuals with one or more comorbidities.

**Implications of all the available evidence**

Available evidence supports the benefits of improving access to regular COVID-19 revaccination for vulnerable subpopulations in LMICs, many of which have discontinued their COVID-19 vaccine booster programmes post-pandemic. Policymakers in LMICs should consider allocating sustainable resources to support COVID-19 vaccine booster programs, especially for vulnerable populations. Future studies with prolonged follow-up are needed to assess the long-term durability of ancestral-strain and strain-adapted vaccines, against potentially emerging SARS-CoV-2 variants with evolving immune-evasive characteristics.

## Introduction

As of September 1st, 2025, COVID-19 caused by SARS-CoV-2 has resulted in approximately 7 million reported deaths worldwide,^1^ with substantial variations in reported case-fatality ratios between geographical settings, in part explained by differences in disease severity, age distribution, pre-existing comorbidities, quality of hospital care received, and existing immunity levels.^2^^,^^3^ Since November 2021, the Omicron variant and its emerging sublineages have been predominant worldwide due to higher transmissibility yet lower disease severity.^2^ Nonetheless, older or immunocompromised individuals and individuals with underlying comorbidities remain at risk of adverse clinical outcomes.

As the pandemic evolved, mass COVID-19 vaccination campaigns based on ancestral-strain vaccines have been effective in preventing SARS-CoV-2-associated severe illness and death worldwide. However, vaccine-elicited protective immunity against infection has been shown to wane within months, compounded by the emergence of new SARS-CoV-2 subvariants that contain immune-evasive escape mutations, warranting regular revaccination to boost immunity.^4^^,^^5^ Studies mostly from high-income countries in North America, Europe and Asia have indicated that ancestral-strain booster vaccines provide moderate protection against severe and fatal outcomes from recent Omicron subvariants like XBB and JN.1, typically waning after six months,^4^^,^^5^ although available data suggest that the protection could be prolonged for high-risk patients.

Indonesia, a middle-income country with the world's fourth largest population (281 million), reported the highest number of COVID-19 cases (6.8 million) and deaths (162,000) in southeast Asia (as per August 1, 2025) through three major waves,^6^ driven by B.1.466.2 (pre-delta), Delta (B.1.617.2, AY.23, and AY.24) and Omicron (B.1.1.529), and three minor waves, driven by Omicron subvariants BA.4/BA.5, XBB and JN.1. Indonesia's vaccination campaign, commenced in January 2021, was mostly based on inactivated whole-virus vaccines (CoronaVac, Sinovac, China) and later viral vector vaccines (ChAdOx1, AstraZeneca, UK), and by January 2023, 86.8% of the population had received primary vaccination.^7^ A hospital-based cohort study in Indonesia, spanning 2020–2023, found that individuals who had received one or more vaccine doses (mostly CoronaVac) six months prior had a 98% reduced risk of severe clinical disease and 56% reduced risk of death from Omicron sub-variants (BA.1/BA.2, BA.4/BA.5 and XBB), compared with unvaccinated individuals.^8^ Since January 2022, the Indonesian government has provided ancestral-strain vaccine booster doses to vulnerable groups, including health care workers (HCWs), individuals aged ≥60 years and immunocompromised people, reaching a general population coverage of 39.1% for the third dose and 2.0% for the fourth dose by March 1, 2025.^7^ However, after declaring the end of the pandemic on June 21, 2023,^9^ the Indonesian government, as in many low- and middle-income countries (LMICs), has discontinued the vaccine booster programme, and updated-strain vaccine boosters have not been introduced to date.

There has been limited evaluation of the longer-term durability of protection of ancestral-strain vaccine boosters against severe and fatal disease outcomes from the latest Omicron subvariants, particularly in settings where there has been limited access to regular revaccination. This real-world study assessed the absolute VE among those who received primary vaccination only, and incremental VE among those who received a third- or fourth-dose booster, against symptomatic infections, hospitalisations and mortality, among the general Indonesian population and high-risk subpopulations, from March 2023 through May 2024.

## Methods

### Study design and population

**Fig. 1:**
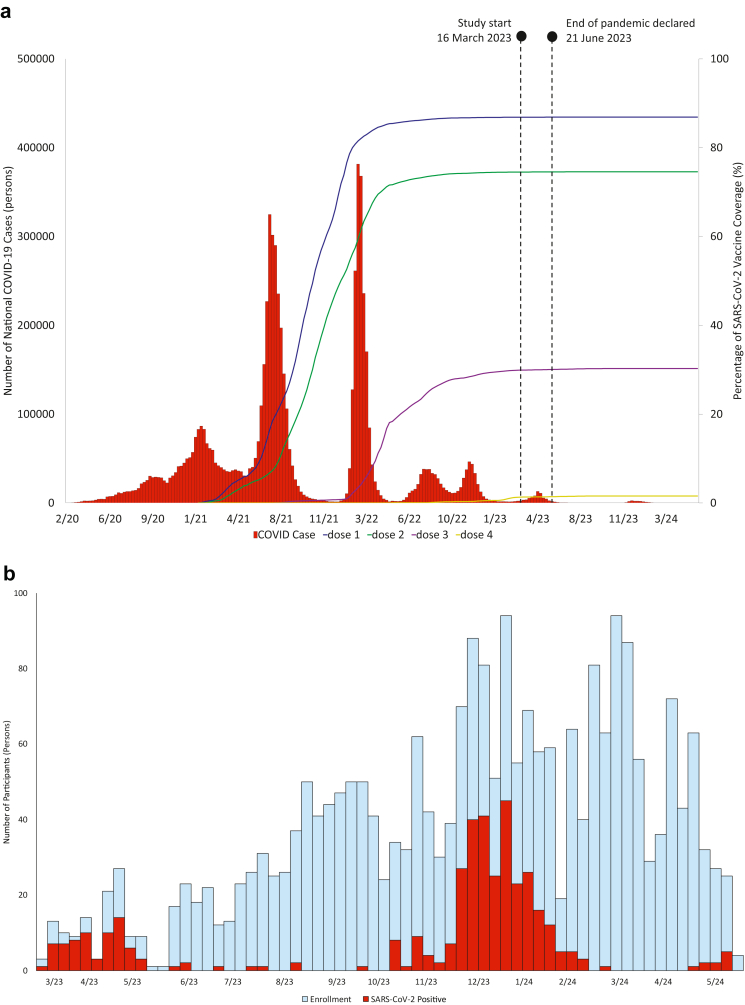
**Timelines of the national SARS-CoV-2 epidemic (a) and study enrollment (b)**. **a**. Timeline of the national SARS-CoV-2 epidemic caused by the successive SARS-CoV-2 variants in Indonesia, from March 2020 through May 2024. The bar chart (left y-axis) shows the number of nationwide weekly new COVID-19 cases. The x-axis shows date in MM/YY format. Indonesia experienced three major SARS-CoV-2 waves, driven by B.1.466.2 (pre-delta), Delta (B.1.617.2, AY.23, and AY.24) and Omicron (B.1.1.529), and three minor waves, driven by Omicron subvariants BA.4/BA.5, XBB and JN.1. The line graph (right y-axis) shows the cumulative percentage of the general population who has received COVID-19 vaccines by dose 1–4. At the start of the study, in the study catchment areas of Jakarta and Yogyakarta provinces, vaccine coverage was 100% and 96% for second vaccine dose, 73.8% and 52% for third dose, and 6.7% and 2.8% for fourth dose, respectively (as per March 15th, 2023). The government of Indonesia declared the end of the COVID-19 pandemic on June 21, 2023, which likely resulted in declining access and willingness to testing and reporting among the general population. **b**. Participants were enrolled between 16 March 2023 and 31 May 2024. The bar graph shows the number of patients enrolled in the cohort (blue bars), including the COVID-19 cases (red bars). During the study period, most COVID-19 cases were enrolled during two SARS-CoV-2 epidemic waves from March through May 2023 (XBB/XBB.1) and from November 2023 through February 2024 (JN.1).

The study was a multicentre, test-negative case–control study, following WHO recommendations.^10^^,^^11^ The catchment areas of Jakarta (national capital city of 11 million inhabitants) and Yogyakarta (provincial capital city of 4 million inhabitants), both located in the island of Java, between the start of the pandemic and the study start, comprised 23.9% of all COVID-19 tests conducted, 5.7% of total reported infections and 13.7% of deaths nationwide.^12^ Fig. 1 provides a national timeline of SARS-CoV-2 waves and vaccine coverage. At the start of the study, in Jakarta and Yogyakarta provinces, vaccine coverage was 100% and 96% for second vaccine dose, 73.8% and 52.5% for third-dose booster, and 6.7% and 2.8% for fourth-dose booster, respectively (as per March 15th, 2023).^7^ The Indonesian government declared the end of the pandemic on June 21, 2023—three months after the start of study recruitment—which likely resulted in an overall reduced access and willingness to COVID-19 testing and reporting among the general population. However, because there was no local or national registry, we could not reliably contextualize any changes in testing practices over time.

The study was conducted at 14 test sites, including seven hospitals, five laboratories and two primary health care centers, which were purposely selected based on the highest test numbers (based on the latest statistics from the province health office), geographic coverage, diversity of patient populations and site willingness. Study staff screened all individuals who sought SARS-CoV-2 testing at the study sites, and consecutively enrolled consenting individuals who met the eligibility criteria, between 16 March 2023 and 31 May 2024, and followed them for up to 30 days for outcome assessment. Inclusion criteria were individuals age 18 years or above, seeking a COVID-19 diagnostic test (rtPCR or antigen rapid diagnostic test [RDT] performed on a nasopharyngeal and/or oropharyngeal swab) because of having acute onset (within the last 10 days) of any one or more signs or symptoms (including fever, cough, general weakness/fatigue, headache, myalgia, sore throat, coryza, dyspnoea, and tachypnoea), and consenting to participate in the study. There were no additional exclusion criteria. Cases were symptomatic individuals who tested SARS-CoV-2 rtPCR or RDT positive at study enrolment. Controls were symptomatic individuals who tested SARS-CoV-2 rtPCR or RDT negative at study enrolment (i.e. had an alternative diagnosis), with RDT-negative participants offered a confirmatory rtPCR test; controls who developed symptoms within 30 days of follow-up, sought client-initiated testing and tested SARS-CoV-2 rtPCR or RDT positive were reclassified as cases and followed up accordingly.

### Data collection

Data were collected through a brief digital questionnaire in REDCap by study staff. At the enrolment visit, we collected demographics (age, sex, place of residence; data verified using national identify card), presenting signs and symptoms, date of onset, pre-existing comorbidities, SARS-CoV-2 infection history (data mostly based on self-report), vaccination history (number of doses received, vaccine type, date of vaccination; data verified using the government vaccination certificate), and current SARS-CoV-2 test results (rtPCR and/or RDT, based on laboratory result). A comorbidity was defined as known chronic heart disease, chronic pulmonary disease, autoimmune disease, chronic liver disease, diabetes mellitus, chronic hematologic disease, hypertension, obesity, active tuberculosis and/or HIV/AIDS. At 30-day follow-up, we collected clinical outcome data on death or alive status (for cases and controls) and disease severity (for cases) through telephone interview or, if non-responding, home visit. Medical information on cause of death and/or hospitalisation was mostly obtained by self-report. Data completeness was high overall (Figure S1). Samples that were rtPCR-confirmed SARS-CoV-2 positive with Ct-value <30 were forwarded to the Genomik Solidaritas Indonesia (GSI) laboratory in Jakarta or Genomic Laboratory at UGM in Yogyakarta for SARS-CoV-2 whole genome sequencing, using established methods.^8^

### Statistical methods

We designed the study to achieve a prespecified precision for the vaccine effectiveness (VE) estimate.^10^^,^^11^ Assuming a VE of 50% for symptomatic COVID-19 after three vaccine doses during Omicron,^13^ 40% third dose coverage, 1:4 case–control ratio, with type 1 error rate of 5%, we estimated a target sample size of 5724 (1431 cases and 4293 controls) to give a precision of ±7%. We used a 14-day cut-off for considering a person protected from vaccination to optimize the validity of the VE estimates. We described participant characteristics using means (SD), medians (IQR) and proportions, as appropriate. We used multivariable logistic regression to estimate vaccine effectiveness (VE) against symptomatic disease, COVID-19-related hospitalisation and death within 30 days as the outcomes of interest and vaccination status as the primary exposure of interest, adjusted for main confounders, i.e. age, sex, previous COVID-19, comorbidities and calendar time (continuous variable, days).

For symptomatic COVID-19, we compared the odds of vaccination in cases compared with that in controls. For hospitalization among cases, we compared the odds of vaccination in hospitalised cases with that in non-hospitalised cases. For death, we compared the odds of vaccination in deceased cases compared with that in alive cases. VE was calculated as (1-aOR) × 100%, estimating absolute (2 vaccine doses vs unvaccinated) and incremental VE (3 vs 2, and 4 vs 3 vaccine doses). To compare estimates, statistical significance was concluded where 95% confidence intervals (CIs) did not overlap. Additional analyses were stratified by presence of comorbidity (yes/no) and age groups (18–50 and > 50 yrs). Stratified analyses for time intervals since most recent vaccination and vaccine types or combinations were not feasible due to limited statistical power. To account for any time imbalance between recruitment of the cases and controls, we conducted a sensitivity analysis that only included the participants who were enrolled during the two SARS-CoV-2 epidemic waves from March through May 2023 (XBB/XBB.1), and from November 2023 through February 2024 (JN.1).

### Ethics statement

This study obtained ethical approval from the Medical and Health Research Ethics Committee (MHREC), Faculty of Medicine, Public Health and Nursing, Universitas Gadjah Mada and Dr. Sardjito General Hospital (KE/FK/0357/EC/2022, KE/FK/0548/EC/2023, and KE/FK/0328/EC/2024), Oxford Tropical Research Ethics Committee (566-22), and US CDC (CGH-VPDST-12/21/22-6b7f3). All participants provided informed consent.

### Role of the funding source

The US Centers for Diseases Control and Prevention funded this study, and were involved in the study design, data interpretation, writing of the report, and decision to submit for publication. The first, second and last authors had full access to all the data and share the final responsibility to submit for publication. The findings and conclusions in this manuscript are those of the authors and do not necessarily represent the official position of the funding agencies.

## Results

### Participant characteristics

**Fig. 2:**
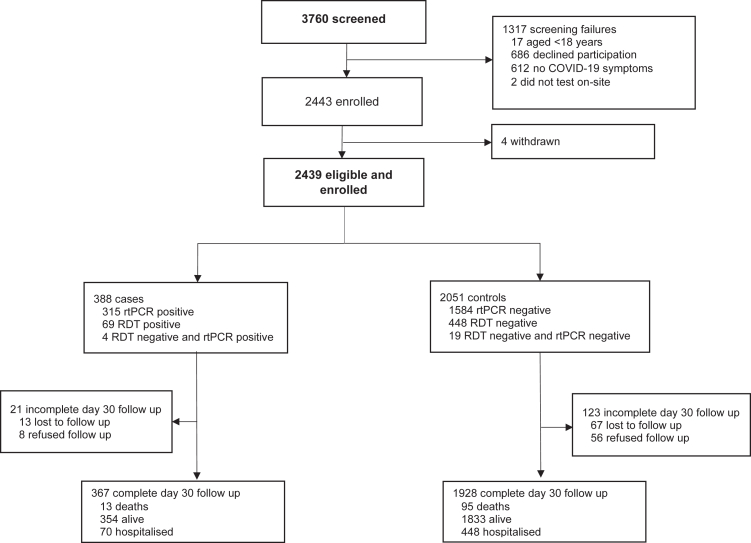
**Study flow diagram**. Participants who had either a positive rtPCR or RDT were classified as cases. Participants who had a negative rtPCR were classified as controls. Participants who had a negative RDT were offered a study-sponsored confirmatory rtPCR (if not accepted were classified as controls). Overall, diagnosis was confirmed with rtPCR for 78.8% (1922/2439) of participants. Abbreviations: RDT, rapid diagnostic test; rtPCR, reverse transcription polymerase chain reaction.

Of 3760 individuals screened for study eligibility, 2439 (65%) were eligible and enrolled, comprising 388 cases and 2051 controls (Fig. 2). Fig. 1 presents a timeline of the national SARS-CoV-2 epidemic and the study enrollment. Although the participants were enrolled throughout the whole study period, most COVID-19 cases were enrolled during two SARS-CoV-2 peaks between March and May 2023 and between November 2023 and February 2024. Diagnosis was ascertained with rtPCR testing for 78.8% (1922/2439) of participants. SARS-CoV-2 vaccine status was verified against the vaccine certificate for 2378 (97.5%) and was self-reported for 61 (2.5%) participants. Among the SARS-CoV-2 sequences obtained from the COVID-19 participants (143, 36.9%), the predominant subvariants were Omicron XBB/XBB.1-like and JN.1 (Figure S2). Women comprised 1371 (56.2%) of the participants. The median age was 35 years (IQR 25–56) overall, including 757 (31.0%) aged 50 years or older. 21.9% (535/2439) of the participants reported one or more known comorbidities, the most prevalent being hypertension, asthma, chronic heart disease and diabetes mellitus (Table 1). Compared to controls, COVID-19 participants were younger, more frequently had a history of COVID-19, more frequently had received a vaccine booster, and received their latest vaccine dose more recently, whereas comorbidities were similarly distributed between the cases and the controls. Of the 367 (94.6%) cases and 1928 (94.0%) controls for whom 30-day outcome data were available, 19.1% (70/367) and 23.2% (448/1928) were hospitalised (p = 0.075) (including ICU admission for 16.3% [60/367] and 0.21% [4/1928]) and 3.5% (13/367) and 4.9% (95/1928) died (p = 0.251), respectively.

**Table 1:** Participant characteristics at enrolment.

Variables	Controls Covid-19 negative (n = 2051)	Cases Covid-19 positive (n = 388)	Overall (n = 2439)	p-value
**Age–median (IQR)**	36 (25–56)	31 (25–52)	35 (25–56)	0.025
**Age groups**				0.118
18–29	793 (38.7%)	173 (44.6%)	966 (39.6%)	
30–39	370 (18.0%)	66 (17.0%)	436 (17.9%)	
40–49	237 (11.6%)	43 (11.1%)	280 (11.5%)	
50–59	214 (10.4%)	43 (11.1%)	257 (10.5%)	
≥60	437 (21.3%)	63 (16.2%)	500 (20.5%)	
**Sex**				0.051
Male	916 (44.7%)	152 (39.2%)	1068 (43.8%)	
Female	1135 (55.3%)	236 (60.8%)	1371 (56.2%)	
**Location**				<0.001
Yogyakarta	1883 (91.8%)	318 (81.9%)	2201 (90.2%)	
Jakarta	168 (8.2%)	70 (18.1%)	238 (9.8%)	
**Previous COVID-19**				<0.001
No	1373 (66.9%)	223 (57.5%)	1596 (65.4%)	
Yes	678 (33.1%)	165 (42.5%)	843 (34.6%)	
**Number and type of vaccine doses**^a^				<0.001
Unvaccinated	230 (11.2%)	33 (8.5%)	263 (10.8%)	
One dose	46 (2.2%)	10 (2.6%)	56 (2.3%)	
CV	19 (0.91%)	6 (1.6%)	25 (1.0%)	
AZ	15 (0.72%)	2 (0.53%)	17 (0.72%)	
Other^b^	12 (0.58%)	2 (0.52%)	14 (0.57%)	
Two doses	419 (20.4%)	58 (14.9%)	477 (19.6%)	
2CV	268 (13.0%)	44 (11.3%)	312 (12.8%)	
2AZ	83 (4.1%)	6 (1.5%)	89 (3.6%)	
2mRNA	24 (1.2%)	2 (0.52%)	26 (1.1%)	
Other^c^	44 (2.2%)	6 (1.6%)	50 (2.1%)	
Three doses	1081 (52.7%)	205 (52.8%)	1286 (52.7%)	
2CV-mRNA	402 (19.6%)	96 (24.8%)	498 (20.4%)	
2CV-AZ	293 (14.3%)	50 (12.9%)	343 (14.1%)	
3AZ	137 (6.7%)	17 (4.4%)	154 (6.3%)	
2AZ-mRNA	94 (4.6%)	21 (5.4%)	115 (4.7%)	
Other^d^	155 (7.5%)	21 (5.4%)	176 (7.2%)	
Four doses	271 (13.2%)	82 (21.1%)	353 (14.5%)	
2CV-2mRNA	178 (8.7%)	62 (15.9%)	240 (9.8%)	
2CV-AZ-mRNA	41 (1.9%)	9 (2.3%)	50 (2.1%)	
3AZ-mRNA	13 (0.62%)	0 (0%)	13 (0.53%)	
Other^e^	39 (1.9%)	11 (2.8%)	50 (2.1%)	
Five doses	4 (0.20%)	0 (0.00%)	4 (0.16%)	
4CV-mRNA	4 (0.20%)	0 (0.00%)	4 (0.16%)	
**Time since most recent vaccination (days)–Median (IQR)**				<0.001
Two doses	832 (723–909)	802 (752–864)	826 (731–905)	
Three doses	600 (498–709)	603 (465–679)	601 (493–701)	
Four doses	407 (314–508)	344 (281–494)	400 (303–498)	
**Number of comorbidities**^f^				0.443
0	1601 (78.1%)	303 (78.1%)	1904 (78.1%)	
1	342 (16.7%)	70 (18.0%)	412 (16.9%)	
2 or more	108 (5.3%)	15 (3.9%)	123 (5.0%)	

Data are presented as N (%) unless otherwise indicated. Cases were defined as symptomatic individuals who had a positive SARS-CoV-2 rtPCR or antigen rapid diagnostic test (RDT) at study enrolment. Controls were defined as symptomatic individuals who tested SARS-CoV-2 rtPCR or RDT negative at study enrollment.

Abbreviations: AZ, AstraZeneca/ChAdOx1; CV, CoronaVac; mRNA, BNT162b2/Pfizer-BioNTech or mRNA-1273/Moderna; UNK, Unknown.

a Vaccine status could be verified for 2378 (97.5%) participants and was self-reported for 61 (2.5%) participants.

b mRNA (n = 12), Sinopharm, Indovax (n = 1 each).

c 2Indovax (n = 22); Biofarma-CV (n = 7); Sinopharm–sinopharm, Indovax-sinopharm (n = 5 each); CV-AZ (n = 4), CV-mRNA, AZ-mRNA (n = 3 each), AZ-CV (n = 1).

d 3mRNA (n = 45); 2Indovax-mRNA, 2Indovax-AZ (n = 17 each); 3CV (n = 16); CV-Indovax-mRNA (n = 11); 3Sinopharm (n = 10); 2mRNA-AZ (n = 8); CV-Indovax-AZ, Indovax-CV-mRNA (n = 6 each); 2CV-Indovax (n = 5); 2CV-Sinopharm, 3UNK (n = 4 each); CV-AZ-mRNA, Indovax-CV-AZ (n = 3 each); 2AZ-Indovax, CV-mRNA-mRNA, AZ-2mRNA, Indovax-2mRNA, 2Sinopharm-mRNA, CV-2AZ, mRNA-CV-mRNA (n = 2 each); 2AZ-CV, mRNA-2AZ, mRNA-AZ-mRNA, 2Indovax-CV, 3Indovax, 2Indovax-Sinopharm, Indovax-2CV, Indovax-CV-Sinopharm, 2CV-UNK (n = 1 each).

e 2AZ-2mRNA (n = 10); 4mRNA (n = 6); 2CV-mRNA-Indovax (n = 4); 4CV, 3CV-mRNA, 2CV-AZ-Indovax (n = 3); CV-Indovax-2mRNA, 3mRNA-AZ (n = 2 each); 3CV-AZ, 2CV-mRNA-Sinopharm, 4AZ, 2CV-2AZ, 2CV-AZ-Sinopharm, 2CV-AZ-Zifivax, 3AZ-Indovax, 2CV-2Indovax, Indovax-2AZ-mRNA, CV-2AZ-mRNA, Indovax-2CV-mRNA, Indovax-CV-2mRNA, Indovax-CV-AZ-mRNA, 2Indovax-2mRNA, 2Indovax-AZ-mRNA, 4Sinopharm, 4UNK (n = 1 each).

f Reported comorbidities included hypertension (n = 329; 13.5%), asthma (n = 199; 8.2%), chronic heart disease (n = 183; 7.5%), diabetes mellitus (n = 178; 7.3%), chronic obstructive pulmonary disease (n = 57; 2.3%), chronic kidney disease (n = 47; 2.0%), tuberculosis (n = 30; 1.2%), obesity (n = 22; 0.9%), chronic haematological disorders (n = 4; 0.2%), chronic liver diseases (n = 3; 0.1%), and HIV/AIDS (n = 2; 0.1%).

### Vaccine effectiveness

**Fig. 3:**
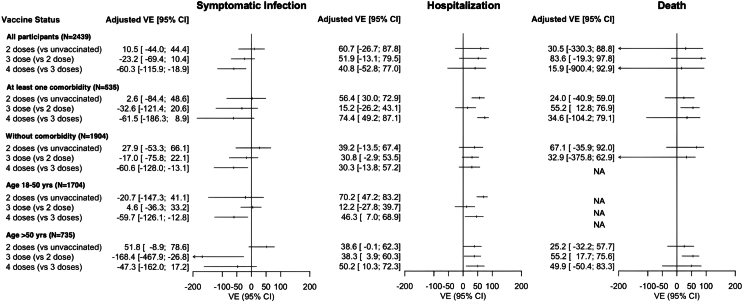
**Forest plots of vaccine effectiveness against SARS-CoV-2 symptomatic infection, hospitalization and death**. Forest plot shows results of multivariable logistic regression models, adjusted for main confounders (age, sex, calendar date, previous COVID-19 and comorbidities). VE was calculated as (1-aOR) × 100%, estimating absolute (unvaccinated vs 2 vaccine doses) and incremental VE (3 vs 2, and 4 vs 3 vaccine doses). Comorbidity was defined as presence of known chronic heart disease, chronic pulmonary disease, autoimmune disease, chronic liver disease, diabetes mellitus, chronic hematology disease, hypertension, obesity, active tuberculosis and/or HIV/AIDS. Abbreviations: NA, not available; VE, vaccine effectiveness.

Table 2 and Fig. 3 present the absolute and relative vaccine effectiveness estimates.

**Table 2:** Vaccine effectiveness against SARS-CoV-2 symptomatic infection, hospitalization and death, overall and stratified for age and comorbid subgroups.

	Symptomatic SARS-CoV-2 infection		COVID-19-related hospitalization		COVID-19-related death	
	Adjusted OR (95% CI)	Adjusted VE (95% CI)	Adjusted OR (95% CI)	Adjusted VE (95% CI)	Adjusted OR (95% CI)	Adjusted VE (95% CI)
**All participants (N = 2439)**						
2 Doses (vs unvaccinated)	0.89 (0.56–1.4)	10.5 (−44.0 to 44.4)	0.39 (0.12–1.2)	60.7 (−26.7 to 87.8)	0.70 (0.11–4.3)	30.5 (−330.3 to 88.8)
3 Dose (vs 2 dose)	1.2 (0.90–1.7)	−23.2 (−69.4 to 10.4)	0.48 (0.20–1.1)	51.9 (−13.1 to 79.5)	0.16 (0.02–1.2)	83.6 (−19.3 to 97.8)
4 Doses (vs 3 doses)	**1.6 (1.2–2.2)**	**−60.3 (−115.9 to −18.9)**	0.59 (0.23–1.5)	40.8 (−52.8 to 77.0)	0.84 (0.07–10)	15.9 (−900.4 to 92.9)
**Participants with at least one comorbidity (N = 535)**						
2 Doses (vs unvaccinated)	0.97 (0.51–1.8)	2.6 (−84.4 to 48.6)	**0.44 (0.27–0.70)**	**56.4 (30.0–72.9)**	0.76 (0.41–1.4)	24.0 (−40.9 to 59.0)
3 Dose (vs 2 dose)	1.3 (0.79–2.2)	−32.6 (−121.4 to 20.6)	0.85 (0.57–1.3)	15.2 (−26.2 to 43.1)	**0.45 (0.23–0.87)**	**55.2 (12.8–76.9)**
4 Doses (vs 3 doses)	1.6 (0.91–2.9)	−61.5 (−186.3 to 8.9)	**0.26 (0.13–0.51)**	**74.4 (49.2–87.1)**	0.65 (0.21–2.0)	34.6 (−104.2 to 79.1)
**Participants without comorbidity (N = 1904)**						
2 Doses (vs unvaccinated)	0.72 (0.34–1.5)	27.9 (−53.3 to 66.1)	0.61 (0.33–1.1)	39.2 (−13.5 to 67.4)	0.33 (0.08–1.4)	67.1 (−35.9 to 92.0)
3 Dose (vs 2 dose)	1.2 (0.78–1.8)	−17.0 (−75.8 to 22.1)	0.69 (0.46–1.0)	30.8 (−2.9 to 53.5)	1.3 (0.37–4.8)	32.9 (−375.8 to 62.9)
4 Doses (vs 3 doses)	**1.6 (1.1–2.2)**	**−60.6 (−128.0 to −13.1)**	0.70 (0.43–1.1)	30.3 (−13.8 to 57.2)	NA	NA
**Participants aged 18–50 yrs (N = 1704)**						
2 Doses (vs unvaccinated)	1.2 (0.59–2.5)	−20.7 (−147.3 to 41.1)	**0.30 (0.17–0.53)**	**70.2 (47.2–83.2)**	NA	NA
3 Dose (vs 2 dose)	0.95 (0.67–1.4)	4.6 (−36.3 to 33.2)	0.88 (0.60–1.3)	12.2 (−27.8 to 39.7)	NA	NA
4 Doses (vs 3 doses)	**1.6 (1.1–2.3)**	**−59.7 (−126.1 to –12.8)**	**0.54 (0.31–0.93)**	**46.3 (7.0–68.9)**	NA	NA
**Participants aged >50 yrs (N = 735)**						
2 Doses (vs unvaccinated)	0.48 (0.21–1.1)	51.8 (−8.9 to 78.6)	0.61 (0.38–1.0)	38.6 (−0.10 to 62.3)	0.75 (0.42–1.3)	25.2 (−32.2 to 57.7)
3 Dose (vs 2 dose)	**2.7 (1.3 to 5.7)**	**−168.4 (−467.9 to −26.8)**	**0.62 (0.40–0.96)**	**38.3 (3.9–60.3)**	**0.45 (0.24–0.82)**	**55.2 (17.7–75.6)**
4 Doses (vs 3 doses)	1.5 (0.83–2.6)	−47.3 (−162.0 to 17.2)	**0.50 (0.28–0.90)**	**50.2 (10.3–72.3)**	0.50 (0.17–1.5)	49.9 (−50.4 to 83.3)

Table shows results of multivariable logistic regression models, adjusted for main confounders (age, sex, calendar date, previous COVID-19 and comorbidities). VE was calculated as (1-aOR) × 100%, estimating absolute (unvaccinated vs 2 vaccine doses) and incremental VE (3 vs 2, and 4 vs 3 vaccine doses). Comorbidity was defined as known chronic heart disease, chronic pulmonary disease, autoimmune disease, chronic liver disease, diabetes mellitus, chronic hematologic disease, hypertension, obesity, active tuberculosis and/or HIV/AIDS.

Bolded values indicate statistical significance was concluded where the 95% confidence limits did not include 1 for OR or 0 for VE.

Abbreviations: NA, not available; OR, odds ratio; VE, vaccine effectiveness.

### Residual protection of two-dose primary vaccination (absolute VE)

Compared to unvaccinated participants, overall, participants who had received two-dose primary vaccination (in the period of 10–40 [median 27] months prior), were not protected against symptomatic infection (absolute VE 10.5%; 95% CI −44.0 to 44.4), hospitalization (60.7%; −26.7 to 87.8) or death (30.5%; −330.3 to 88.8). These results were similar for participants aged 50 years or older (51.8%; −8.9 to 78.6; 38.6%; −0.10 to 62.3; 25.2%; −32.2 to 57.7, respectively). However, participants with one or more comorbidities were moderately protected against hospitalization (56.4%; 30.0–72.9), but not against symptomatic infection (2.6%; −84.4 to 48.6) or death (24.0%; −40.9 to 59.0).

### Incremental protection of third-dose booster

Compared to participants who received two-dose primary vaccination, overall, participants who had received a third-dose booster (in the period of 1–33 [median 20] months prior) were not incrementally protected against symptomatic infection (incremental VE −23.2%; −69.4 to 10.4), hospitalization (51.9%; −13.1 to 79.5) or death (83.6%; −19.3 to 97.8). Participants aged 50 years or older were incrementally protected against hospitalization (38.3%; 3.9–60.3) and death (55.2%; 17.7–75.6), although they had an increased risk of symptomatic infection (incremental VE was negative; −168.4%; −467.9 to −26.8). Participants with one or more comorbidities were incrementally protected against death (55.2%; 12.8–76.9), but not against symptomatic infection (−32.6%; −121.4 to 20.6) or hospitalization (15.2%; −26.2 to 43.1).

### Incremental protection of fourth-dose booster

Compared to participants who received three vaccine doses, overall, participants who had received a fourth-dose booster (in the period of 1–26 [median 13] months prior) had an increased risk of symptomatic infection (incremental VE was negative; −60.3%; −115.9 to −18.9), and were not incrementally protected against hospitalisation (40.8%; −52.8 to 77.0) and death (15.9%; −900.4 to 92.9). Participants aged 50 years or older were incrementally protected against hospitalization (50.2%; 10.3–72.3), but not against symptomatic infection (−47.3%; −162.0 to 17.2) or death (49.9%; −50.4 to 83.3). Participants with one or more comorbidities were incrementally protected against hospitalization (74.4%; 49.2–87.1), but not against symptomatic infection (−61.5%; −186.3 to 8.9) or death (34.6%; −104.2 to 79.1).

### Sensitivity analysis

Restricting the analysis to the participants (364 cases and 724 controls) enrolled during the two major SARS-CoV-2 epidemic waves yielded the same direction and similar effect sizes for the key associations as those identified in the main analysis, although some were no longer statistically significant due to reduced statistical power (Table S1).

## Discussion

This multicentre test-negative case–control study, comprising 2443 symptomatic adults in two major cities in Indonesia, evaluated the longer-term durability of protection from the third- and fourth-dose (homologous or heterologous) boosters of ancestral-strain vaccines. Most COVID-19 cases were enrolled during two SARS-CoV-2 epidemic waves from March through May 2023 (XBB/XBB.1), and from November 2023 through February 2024 (JN.1).

In our study population, primary vaccination with two-dose CoronaVac or ChAdOx1, administered median 27 months prior, was found to provide no residual protection against XBB/JN.1 symptomatic infection, hospitalisation or death, with the notable exception of individuals with one or more comorbidities, who maintained moderate protection against hospitalisation (absolute VE 56.4% [30.0–72.9]), compared to unvaccinated individuals. This findings is largely consistent with most prior studies, which demonstrated waning protection against infection within 6–12 months.^14^^,^^15^

We observed durable, moderate protection from the ancestral-strain vaccine boosters against severe and fatal outcomes from XBB/JN.1 among high-risk groups. The incremental VE of the third-dose booster (mostly homologous ChAdOx1, or heterologous ChAdOx1/mRNA) after a median interval of 20 months was, among older adults, 55.2% (17.7–75.6) against COVID-19-related death and 38.3% (3.9–60.3) against COVID-19-related hospitalisation, and, among individuals with one or more comorbidities, 55.2% (12.8–76.9) against death. Incremental VE of the fourth-dose booster (mostly heterologous mRNA) after a median interval of 13 months was 50.2% (10.3–72.3) against COVID-19-related hospitalisation among older adults and 74.4% (49.2–87.1) among comorbid individuals, but was not protective against death. This finding contrasted with the, seemingly counterintuitive, observation that the odds of symptomatic infection were higher after the fourth-dose booster, compared with after the third-dose booster. This association was likely confounded by the high probability of virus exposure between the third and fourth dose vaccination rounds (JN.1 wave, from November 2023 through February 2024), possibly aggravated by the increasing degrees of immune evasion of recently emerged Omicron subvariants.^16^^,^^17^

Our findings are consistent with previous studies that demonstrated that ancestral-strain heterologous mRNA or viral vector boosters did not prevent Omicron infections, due to immune waning as well as immune evasion by recent Omicron variants.^4^^,^^18^, ^19^, ^20^ However, previous studies also found that both homologous (mRNA or CoronaVac)^21^, ^22^, ^23^ and heterologous (ChAdOx1/mRNA)^21^^,^^23^^,^^24^ regimens of ancestral-strain monovalent vaccine boosters provided moderate, yet short-lived, protection against severe or fatal outcomes from Omicron infections, typically waning within 4–7 months. The present study adds to this existing evidence suggesting that the protective effects against severe and fatal disease for older adults and individuals with comorbidities, may be sustained for up to 20 months after the third-dose booster and up to 13 months after the fourth-dose booster. The observed sustained protection in high-risk groups may reflect combined effects of recent virus exposure during the XBB and/or JN.1 waves, which can boost neutralising antibodies and maintain broadly cross-reactive T cell responses over time, as well as pre-existing immunity and differential immune system responses in these populations, although data have suggested that protection induced by Omicron infection diminishes within a year.^16^^,^^17^

In addition to strong available evidence of vaccine-induced protection against acute health consequences of COVID-19, available data also support the effectiveness of COVID-19 primary vaccination and booster doses in reducing the risk of post-COVID-19 condition, which could be more pronounced among people aged 65 year or older and patients with multimorbidity.^25^

Since 2022, vaccines containing different SARS-CoV-2 variants have been developed to induce broader immune responses and provide enhanced protection against severe outcomes, initially containing BA.1/BA.4/5 and more recently XBB and JN.1.^26^, ^27^, ^28^ A meta-analysis demonstrated that bivalent mRNA booster doses (administered as the third or subsequent dose) provided about 30–50% VE against infection or symptomatic infection with Omicron subvariants, and 60–70% VE against severe clinical outcomes across all age groups, compared to monovalent ancestral-strain dose regimens.^13^ Recent studies have extended this evidence to XBB.^29^^,^^30^ Consequently, many high-income countries have transitioned to deploying bivalent or subvariant-adapted monovalent boosters, although this has not yet been implemented in Indonesia or most other LMICs.

The study had several strengths. First, the inclusion of different types of health facilities located across two major Indonesian cities allowed representation of diverse patient subpopulations and early detection of emerging subvariants. Second, the test-negative design addressed unmeasured confounding due to health care-seeking behavior and exposure to infectious diseases, as both cases and controls were required to have symptoms in order to be eligible for study inclusion.^11^ Third, we ascertained vaccination status through vaccination cards and medical records, which likely limited exposure misclassification. Lastly, nearly 80% of participants received a SARS-CoV-2 RT-PCR test, which limited the risk of case–control misclassification. Nonetheless, some incidental COVID-19 cases may have been missed by RDT only and therefore misclassified as non-COVID-19 controls, potentially leading to an underestimation of VE.

There are some study limitations. First, although VE estimates were adjusted for the main confounding factors, there may be residual confounding by factors related to severe or fatal COVID-19 outcomes that could not be captured or accounted for (e.g. the effects of missed prior SARS-CoV-2 infection and related immunity levels, or undiagnosed pre-existing underlying conditions). For some variables (e.g. prior infection, comorbidities) we had to rely on self-reporting for most participants, which has potential for recall bias. Second, study recruitment was lower than anticipated, due to an overall decline in COVID-19 cases and the government of Indonesia declaring the end of the pandemic on June 21, 2023. The limited sample size reduced precision of the VE estimates (from ±7% prespecified to ±12% in post-hoc estimation), and precluded some planned stratified analyses (e.g. specific chronic conditions, vaccine regimens and immunity waning). The point estimates should therefore be interpreted with caution. Third, although this analysis reported real-world findings in a varied, urban adult population sample, we recognize that 90% of participants were from a single city (Yogyakarta). The study population was relatively young, which is associated with a lower risk of adverse COVID-19 outcomes. Therefore, the study population may not entirely reflect the rates and risk factors of severe and fatal outcomes in the Indonesian general population, given substantial variations in access to health care services, health seeking behaviour and background health risks. Lastly, we cannot rule out possible confounding by higher uptake or prioritisation of vaccine boosters among higher-risk groups (e.g. older individuals with comorbidities), which could have led to overestimating the effect of vaccination.^9^

In conclusion, this study extends the limited available evidence from LMICs on the durability of protection from ancestral-strain booster vaccine doses. The data support the need for improved access to regular vaccine boosters, for older and comorbid subpopulations in Indonesia. There is also a need for more real-world data with prolonged follow-up to assess the durability of ancestral-strain vaccines against emerging SARS-CoV-2 variants, in order to inform optimal vaccine booster strategies. Context-specific cost-effectiveness analyses to determine the minimal revaccination intervals, populations to be prioritised and the added value of implementing strain-updated vaccine boosters, will be critical for health policy makers in LMICs to guide the allocation of scarce public health resources.

## Contributors

BSW and RLH were the principal investigators, and conceptualised the study with support from VO, RD, AWM, MH, FW and BD. MH, HD, FW and RLH obtained the funding. BSW, VO, KI, MH, AWM, HD, RD, MS, S, AP, DUN, AK, A, MMT and ISB established the cohort, and supervised data collection. AWM and ISB contributed to data verification. BSW, MS, AWM performed the statistical analysis and data visualisations, with critical input from HS, BD and RLH. AP and S conducted and supervised the SARS-CoV-2 whole genome sequencing. BSW, VO, MMT and RLH drafted the manuscript, with critical input from FW, RM, HS and BD. BSW and AWM had full access to all of the data in the study and took responsibility for the integrity of the data and the accuracy of the data analysis. All authors critically revised the manuscript for important intellectual content and all authors gave final approval for this version to be published.

## Data sharing statement

After publication, the datasets used for this study will be made available to others on reasonable request to the corresponding authors. Deidentified participant data will be provided after written approval from the principal investigators.

## Declaration of interests

The authors declare no competing interests.

## Appendix A Supplementary data

Supplementary Material

Translated Abstract
