# Supplementary material for: Durability of protection of ancestral-strain COVID-19 third- and fourth-dose vaccine boosters against Omicron XBB/XBB.1 and JN.1 symptomatic infection, hospitalisation and mortality in Indonesian adults (2023–2024): a test-negative case–control study
Source: Lancet Reg Health Southeast Asia. 2025 Nov 1;42:100689. doi: 10.1016/j.lansea.2025.100689 (PMC12603757; doi:10.1016/j.lansea.2025.100689)
Supplement: Supplementary Material [file mmc1.docx]

**SUPPLEMENTARY MATERIAL**

**Figure S1. Data completeness flow chart**


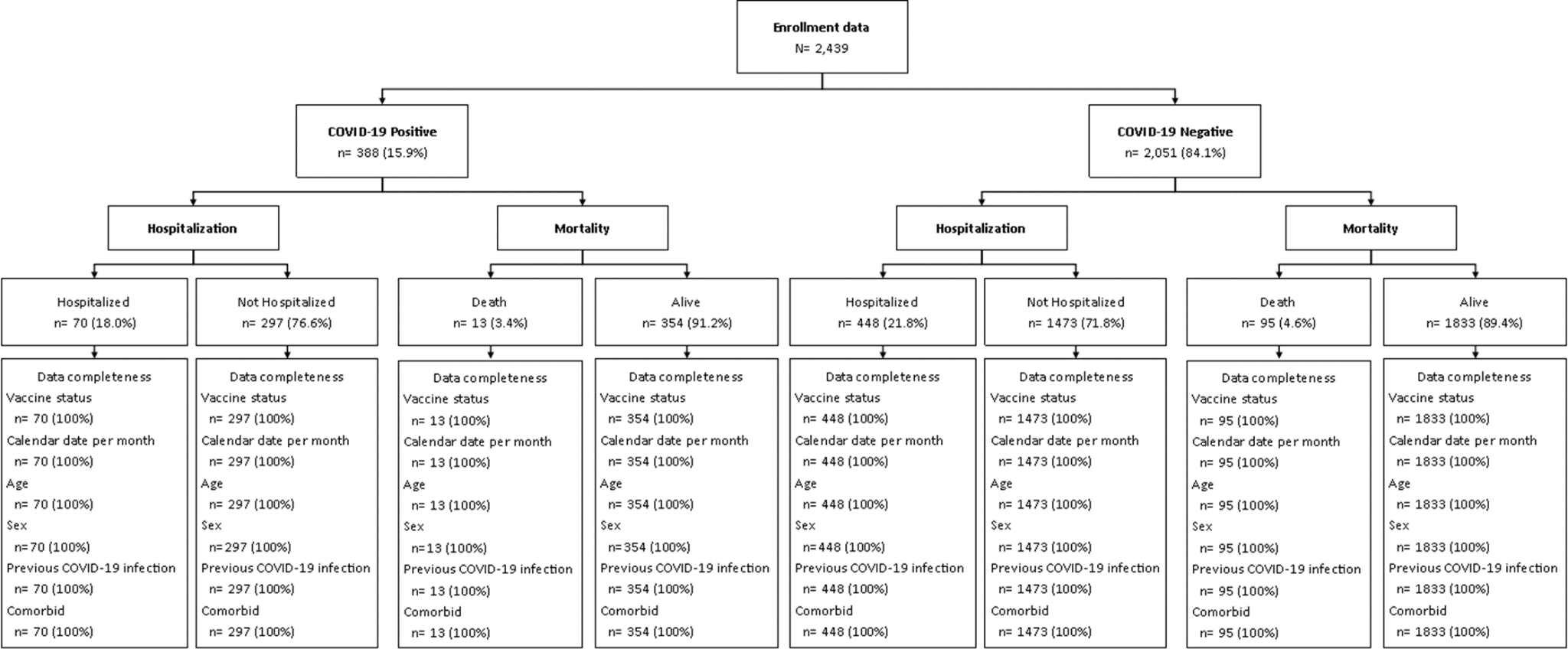


Outcome data were missing for hospitalisation (n=21 cases and n=130 controls)) and mortality (n=21 cases and n=123 controls), also refer to Figure 2.

**Figure S2. Proportions of SARS-CoV-2 variant lineages detected among the participants, from March 2023 through May 2024**


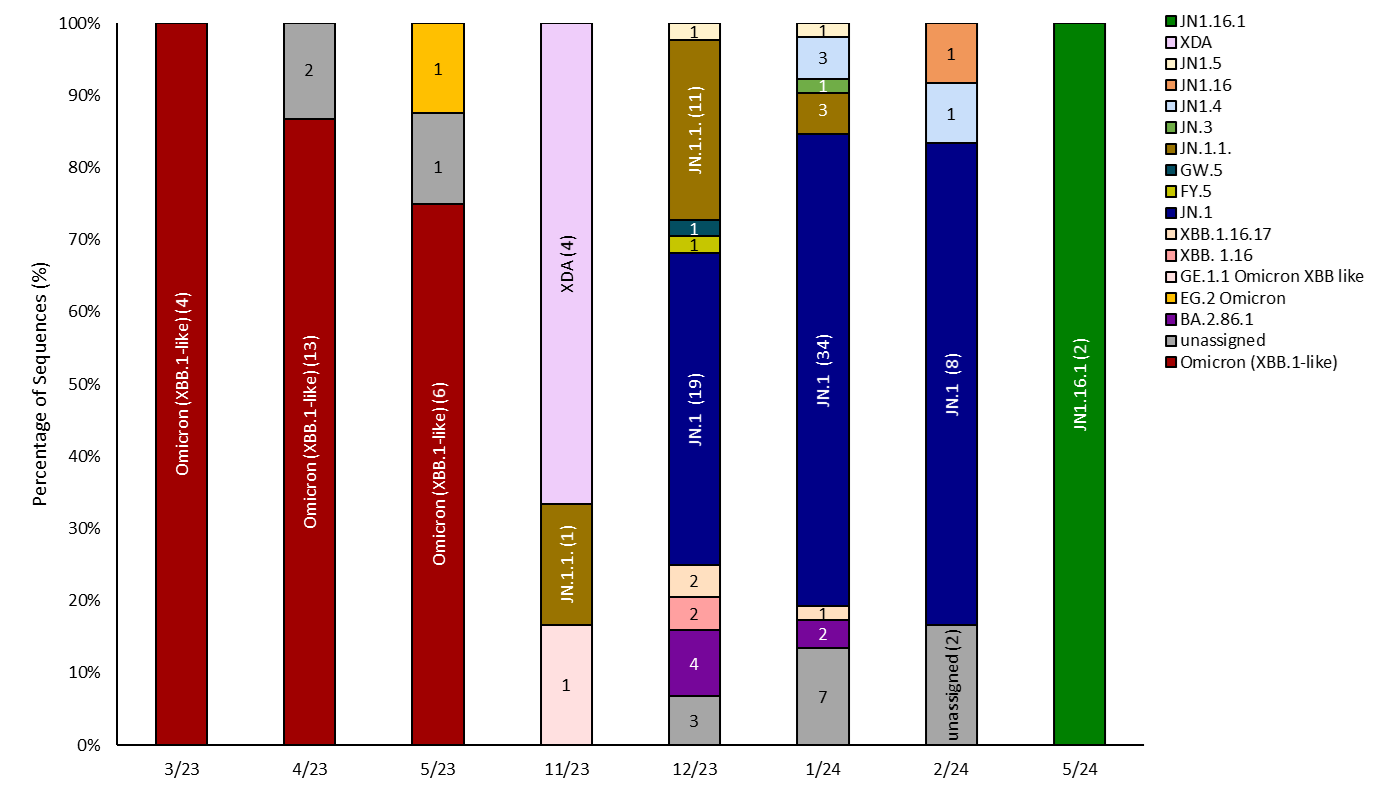


Nasopharyngeal and/or oropharyngeal swabs with positive SARS-CoV-2 RT-PCR with Ct value <30 were submitted to Genomik Solidaritas Indonesia (GSI) Lab in Jakarta or UGM laboratory in Yogyakarta. SARS-CoV-2 could be amplified from 147 samples out of 153 collected samples (96.1%), and 143 of those (97.3%) were successfully sequenced. Briefly, RNA was extracted and converted into cDNA using Lunascript RT supermix and reverse transcriptase enzyme. The ARTIC Network and Midnight protocol were used for library preparation. Libraries were multiplexed on FLO-MIN106 flowcells and sequences were generated on the Oxford Nanopore Technology (ONT) GridION X5. Basecalling was done using MinKnow and Medaka method, followed by Pangolineage assignment of SARS-CoV-2 variants. All SARS-CoV-2 whole genome sequences were reported to the Indonesian Ministry of Health and uploaded to GISAID database. SARS-CoV-2 variants are based on Pangolin lineages and NextStrain SARS-CoV-2 clade. JN.1 like (JN.1 61; JN.1.1 15; FY.5 1; GW.5 1; JN.3 1; JN.1.16 3; JN.1.5 2; JN.1.4 4); XBB.1 like (XBB.1.16.17 3; XBB.1.16 2); BA.2.86.1 6; GE.1.1 (XBB like 1), EG.2 Omicron 1; XBB.1 like 23); XDA 4; Unassigned 15

**Table S1. Sensitivity analysis of vaccine effectiveness, restricted to time periods of the two major SARS-CoV-2 epidemic waves**

|  | **Symptomatic SARS-CoV-2 infection** | | **COVID-19-related hospitalization** | | **COVID-19-related death** | |
| --- | --- | --- | --- | --- | --- | --- |
|  | **Adjusted OR**  **(95% CI)** | **Adjusted VE**  **(95% CI)** | **Adjusted OR**  **(95% CI)** | **Adjusted VE**  **(95% CI)** | **Adjusted OR**  **(95% CI)** | **Adjusted VE**  **(95% CI)** |
| **All participants** |  |  |  |  |  |  |
| 2 doses (vs unvaccinated) | 1.2 (0.7 to 2.0) | -18.0 (-97.4 to 29.4) | 0.6 (0.4 to 1.0) | 38.8 (-4.0 to 64.1) | 0.8 (0.4 to 1.8) | 17.6 (-81.7 to 62.7) |
| 3 dose (vs 2 dose) | 1.2 (0.8 to 1.7) | -18.0 (-68.0 to 17.1) | 0.7 (0.5 to 1.1) | 27.8 (-8.8 to 52.0) | **0.4 (0.2 to 0.9)** | **61.4 (9.4 to 83.6)** |
| 4 doses (vs 3 doses) | **1.6 (1.1 to 2.2)** | **-55.8 (-121.4 to -9.6)** | **0.4 (0.2 to 0.8)** | **56.4 (23.8 to 75.0)** | 0.5 (0.1 to 2.2) | 52.9 (-120.8 to 90.0) |
| **Participants with at least one comorbidity** | | | | | | |
| 2 doses (vs unvaccinated) | 1.2 (0.7 to 2.0) | -17.3 (-96.4 to 29.9) | 0.6 (0.4 to 1.0) | 40.2 (-1.4 to 64.7) | 0.8 (0.4 to 1.8) | 19.0 (-78.4 to 63.2) |
| 3 dose (vs 2 dose) | 1.1 (0.8 to 1.6) | -10.4 (-57.7 to 22.7) | 0.7 (0.4 to 1.0) | 34.1 (1.3 to 56.0) | **0.4 (0.2 to 0.9)** | **63.3 (14.4 to 84.2)** |
| 4 doses (vs 3 doses) | **1.6 (1.1 to 2.3)** | **-62.3 (-131.9 to -13.6)** | **0.4 (0.2 to 0.7)** | **58.3 (27.5 to 76.0)** | 0.4 (0.1 to 2.1) | 56.0 (-105.4 to 90.6) |
| **Participants without comorbidity** | | | | | | |
| 2 doses (vs unvaccinated) | 1.3 (0.7 to 2.1) | -26.3 (-113.6 to 25.3) | 0.6 (0.3 to 1.0) | 41.6 (1.0 to 65.6) | 0.8 (0.4 to 1.8) | 18.6 (-79.4 to 63.1) |
| 3 dose (vs 2 dose) | 1.1 (0.8 to 1.6) | -8.2 (-55.4 to 24.7) | 0.7 (0.5 to 1.1) | 29.8 (-5.1 to 53.1) | **0.4 (0.2 to 0.8)** | **63.6 (15.1 to 84.4)** |
| 4 doses (vs 3 doses) | **1.5 (1.0 to 2.1)** | **-47.6 (-114.4 to -1.6)** | **0.4 (0.2 to 0.7)** | **59.4 (29.5 to 76.7)** | NA | NA |
| **Participants aged 18 to 50 yrs** | | | | | | |
| 2 doses (vs unvaccinated) | 1.3 (0.8 to 2.2) | -34.6 (-123.5 to 18.9) | **0.4 (0.2 to 0.7)** | **59.1 (32.3 to 75.3)** | NA | NA |
| 3 dose (vs 2 dose) | 1.1 (0.8 to 1.6) | -13.8 (-62.2 to 20.1) | 0.8 (0.5 to 1.1) | 23.7 (-13.1 to 48.5) | NA | NA |
| 4 doses (vs 3 doses) | **1.5 (1.1 to 2.1)** | **-49.3 (-112.2 to -5.0)** | **0.6 (0.3 to 0.97)** | **43.4 (3.0 to 67.0)** | NA | NA |
| **Participants aged >50 yrs** | | | | | | |
| 2 doses (vs unvaccinated) | 1.3 (0.8 to 2.2) | -30.8 (-118.2 to 21.6) | **0.4 (0.2 to 0.7)** | **58.8 (31.7 to 75.2)** | **0.5 (0.2 to 0.9)** | **53.0 (1.0 to 77.6)** |
| 3 dose (vs 2 dose) | 1.0 (0.7 to 1.5) | -3.4 (-49.3 to 28.4) | 0.8 (0.6 to 1.2) | 17.5 (-22.6 to 44.5) | 0.4 (0.2 to 1.0) | 55.0 (-1.6 to 80.1) |
| 4 doses (vs 3 doses) | **1.5 (1.0 to 2.2)** | **-50.8 (-119.7 to -3.5)** | **0.5 (0.3 to 0.9)** | **46.0 (7.0 to 68.6)** | 0.6 (0.1 to 2.9) | 36.8 (-189.6 to 86.2) |

Table presents results of multivariable logistic regression models, adjusted for main confounders (age, sex, calendar date, previous COVID-19 and comorbidities). VE was calculated as (1-aOR) x 100%, estimating absolute (unvaccinated versus 2 vaccine doses) and incremental VE (3 versus 2, and 4 versus 3 vaccine doses).

The sensitivity analysis included 364 cases and 724 controls enrolled during the two SARS-CoV-2 epidemic waves from March through May 2023 (XBB/XBB.1), and from November 2023 through February 2024 (JN.1).

Abbreviations: NA, not available; OR, odds ratio; VE, vaccine effectiveness
