# Supplementary material for: Durability of protection of ancestral-strain COVID-19 third- and fourth-dose vaccine boosters against Omicron XBB/XBB.1 and JN.1 symptomatic infection, hospitalisation and mortality in Indonesian adults (2023–2024): a test-negative case–control study
Source: Lancet Reg Health Southeast Asia. 2025 Nov 1;42:100689. doi: 10.1016/j.lansea.2025.100689 (PMC12603757; doi:10.1016/j.lansea.2025.100689)
Supplement: Translated Abstract [file mmc2.docx]

## *This translation in Indonesian bahasa was submitted by the authors and we reproduce it as supplied. It has not been peer reviewed. Our editorial processes have only been applied to the original abstract in English, which should serve as reference for this manuscript.*

## **RINGKASAN**

**Latar belakang:** Vaksin galur awal SARS-CoV-2 terbukti efektif menurunkan angka keparahan penyakit dan kematian akibat SARS-CoV-2 di seluruh dunia. Namun, seiring berjalannya waktu, imunitas yang terbentuk mengalami penurunan sehingga diperlukan vaksinasi tambahan untuk meningkatkan imunitas. Indonesia, seperti kebanyakan negara berpendapatan rendah dan menengah lainnya, tidak menerapkan kebijakan pemberian vaksin tambahan secara rutin pascapandemi. Studi ini menguji ketahanan jangka panjang perlindungan dari vaksin tambahan galur awal (dosis ketiga dan keempat)

**Metode:** Kami melakukan studi kasus-kontrol uji-negatif pada orang dewasa bergejala yang menjalani pemeriksaan SARS-CoV-2 di 14 lokasi tes yang dipilih berdasarkan tujuan tertentu di kota besar Yogyakarta dan Jakarta (Maret 2023–Mei 2024). Individu dengan hasil tes positif didefinisikan sebagai kasus, sedangkan yang negatif sebagai kontrol. Varian SARS-CoV-2 diidentifikasi melalui sekuensing genom utuh. Kami menggunakan regresi logistik multivariat untuk mengestimasi efektivitas vaksin (EV) absolut atau tambahan terhadap infeksi simptomatik serta rawat inap atau kematian terkait COVID-19, dengan penyesuaian terhadap perancu utama.

**Hasil:** Dari 2439 peserta (usia median 35 tahun, 56,2% perempuan), 388 adalah kasus dan 2051 kontrol. Vaksinasi dengan dua dosis primer, dosis ketiga, atau dosis keempat tidak memberikan perlindungan berkelanjutan terhadap infeksi simptomatik Omicron XBB/JN.1 hingga masing-masing median 27, 20, atau 13 bulan pasca vaksinasi . Namun, terdapat perlindungan tambahan dari dosis ketiga (median 20 bulan sebelumnya) terhadap rawat inap (EV 38,3% [95%CI 3,9–60,3]) dan kematian (55,2% [17,7–75,6]) pada kelompok usia lanjut (>50 tahun), serta terhadap kematian (55,2% [12,8–76,9]) pada individu dengan ≥1 komorbiditas. Dosis keempat juga memberikan perlindungan tambahan (median 13 bulan sebelumnya) terhadap rawat inap pada kelompok usia lanjut (50,2% [10,3–72,3]) dan individu dengan ≥1 komorbiditas (74,4% [49,2–87,1]).

**Interpretasi:** Vaksin penguat galur awal memberikan perlindungan sedang dan tahan lama terhadap luaran berat atau fatal akibat infeksi Omicron XBB/JN.1 pada kelompok usia lanjut dan individu dengan komorbiditas. Temuan ini menekankan pentingnya peningkatan akses vaksinasi ulang bagi kelompok rentan di Indonesia.

**Pendanaan:** *US Centers for Disease Control and Prevention*
